# *Antimicrobial Stewardship and Healthcare Epidemiology* year in review, 2022: Celebrating successes while focusing on the future

**DOI:** 10.1017/ash.2022.373

**Published:** 2023-01-11

**Authors:** Priya Nori, Anucha Apisarnthanarak, Pamela L. Bailey, Bradley J. Langford, Lindsay MacMurray, Alexandre R. Marra, Kelly L. Matson, Kari A. Simonsen, Pranavi Sreeramoju, Gonzalo M. Bearman

**Affiliations:** 1 Division of Infectious Diseases, Department of Medicine, Montefiore Health System, Albert Einstein College of Medicine, Bronx, New York, United States; 2 Infectious Diseases Division, Thammasat University Hospital, Pathum Thani, Thailand; 3 Infectious Diseases, Prisma Health, Columbia, South Carolina, United States; 4 Dalla Lana School of Public Health, University of Toronto & Hotel Dieu Shaver Health and Rehabilitation Centre, St. Catharines, Canada; 5 Society for Healthcare Epidemiology of America, Arlington, Virginia, United States; 6 Hospital Israelita Albert Einstein, São Paulo, SP, Brazil; 7 Department of Internal Medicine, University of Iowa Carver College of Medicine, Iowa City, Iowa, United States; 8 College of Pharmacy, University of Rhode Island, Providence, Rhode Island, United States; 9 University of Nebraska Medical Center, Omaha, Nebraska, United States; 10 Jefferson Health, Philadelphia, Pennsylvania, United States; 11 Division of Infectious Diseases, Virginia Commonwealth University Health, Virginia Commonwealth University, Richmond, Virginia, United States

As 2022 ends, we reflect on recent accomplishments and future directions of *Antimicrobial Stewardship and Healthcare Epidemiology* (ASHE). Launched in 2021, the journal is evolving by leaps and bounds. Major accomplishments include a continued growth of submissions, publications, downloads, social media engagements, and the launch of the ASHE podcast. Finally, the journal successfully achieved SCOPUS and PubMed indexing in an expedited fashion.

The overarching theme of our past and present vision is sustained expansion of ASHE on all fronts. We plan to achieve this through the publication of new perspectives, growth in diversity of authors, improvements in author experience via shortened manuscript acceptance and publication times, enhanced social media presence with visual abstracts, and promotion of the ASHE blog and podcast.

In terms of overall submissions, in 2022 we received 239 new manuscripts. We also published the SHEA Spring 2022 proceedings. The number of article downloads significantly increased from 22,311 to 109,223. This growth is due to the enthusiasm of the SHEA membership and the online accessibility of our publications by the international infection prevention and antimicrobial stewardship community. Some of our most read articles in 2022 with the highest Altmetric scores include “The effectiveness of coronavirus disease 2019 (COVID-19) vaccine in the prevention of post–COVID-19 conditions: A systematic literature review and meta-analysis“ by Dr. Alexandre Marra et al, and “Fluoroquinolone stewardship at a community health system: A decade in review“ by Dr. Elena Swingler et al.

Also in 2022, Dr. Pamela Bailey joined us as the social media editor, promoting cutting-edge primary research articles, visual abstracts, and opinion pieces, which were retweeted and cited numerous times. On Twitter, @ASHE_journal surpassed 1,000 followers during IDWeek 2022 and overall followers have doubled since she joined the team. Twitter impressions range between 40,000 and 70,000 per month, with engagements leading to significant increases in link clicks and overall readership of ASHE content. Every paper published in ASHE is tweeted from @ASHE_journal, further promoting our high-quality, open-access content.

We sought new horizons by expanding partnerships with the Society of Infectious Diseases Pharmacists (SIDP), successfully recruiting Dr. Bradley Langford as our newest Associate Editor. We also partnered with the Asia Pacific Society of Infection Control (APSIC), whose 2022 meeting proceedings will soon be published in ASHE.

In addition, we are particularly proud of our novel “Women of Epidemiology” section, a nod to the popular SHEA Spring session created by celebrated female-identifying SHEA leaders. Using “Women in Epi” as a vehicle, we also launched the ASHE podcast to host in-depth conversations with the authors of our most impactful articles.

Dr. Suzanne Bradley, emeritus Editor in Chief of our companion journal*, Infection Control and Hospital Epidemiology*, was the inaugural “Women in Epi” contributor and first podcast guest. She shared her career highlights, challenges, important lessons learned, and her perspectives on our unique role as advocates for healthcare rights of our patients. Later in the episode, Dr. Pamela Bailey spoke of her motivation to write “Restricted Reproductive Health and Infectious Diseases Outcomes: A Call to Action,” which was one of ASHE’s most widely shared and downloaded articles of 2022. This piece was endorsed by the SHEA Diversity, Equity, and Inclusion committee and Board of Trustees in their statement regarding the Dobbs decision. We are proud that ASHE has become a platform to discuss intersections of important issues of our day. Future “Women in Epi” contributors include some of our most treasured colleagues, like Dr. Judy Guzman-Cottrill, who will share with readers her important lessons learned forging her own path in public health and epidemiology.

In November, we celebrated World Antibiotic Awareness Week in style with a publication led by Drs. Bradley Langford, Khalid ElJaaly, and Kelly Matson entitled “Ten ways to Make the Most of World Antimicrobial Awareness Week,” which was also among our most engaging articles of 2022. We had a blast interviewing the authors for our second podcast episode and covered many topics, including the future of social media as a vehicle for stewardship dissemination.

In the upcoming year, we will continue to focus on growth of content, increased downloads, and providing a platform for unique perspectives and high-quality science. Doing so will require the relentless recruitment of first-rate submissions. In addition, we will seek new international partnerships to add a diversity of authors and perspectives to ASHE. We will continue to focus on further improving the ASHE author experience by decreasing the time to first decision and publication, by further enhancing social media promotion, and by providing guidance and assistance with visual abstracts. Podcasts will continue to strategically highlight ASHE content. We will work closely with all authors from low- and middle-income countries to minimize article processing charge barriers to publication. We will continue to publish proceedings from SHEA spring and other cutting-edge science. Our aim is to position ASHE as a premier, fully open-access journal advancing the fields of antimicrobial stewardship and healthcare epidemiology across the globe.

